# Effects of Antibiotics upon the Gut Microbiome: A Review of the Literature

**DOI:** 10.3390/biomedicines8110502

**Published:** 2020-11-16

**Authors:** Theocharis Konstantinidis, Christina Tsigalou, Alexandros Karvelas, Elisavet Stavropoulou, Chrissoula Voidarou, Eugenia Bezirtzoglou

**Affiliations:** 1 Laboratory of Microbiology, Medical School, Democritus University of Thrace, Dragana, 68100 Alexandroupolis, Greece; theoxari_ko@yahoo.gr (T.K.); or ctsigalo@med.duth.gr (C.T.); alexnet.gr@gmail.com (A.K.); 2 Centre Hospitalier Universitaire Vaudois (CHUV), Rue du Bugnon, Vaud, CH-1011 Lausanne, Switzerland; elisabeth.stavropoulou@gmail.com; 3 Public Health Laboratory, Arta Prefecture, 47100 Arta, Greece; xvoidarou@yahoo.gr; 4 Laboratory of Hygiene and Environmental Protection, Medical School, Democritus University of Thrace, 68100 Alexandroupolis, Greece

**Keywords:** antibiotics, resistance, gut microbiome, microbiota

## Abstract

The human gastrointestinal tract carries a large number of microorganisms associated with complex metabolic processes and interactions. Although antibiotic treatment is crucial for combating infections, its negative effects on the intestinal microbiota and host immunity have been shown to be of the utmost importance. Multiple studies have recognized the adverse consequences of antibiotic use upon the gut microbiome in adults and neonates, causing dysbiosis of the microbiota. Repeated antibiotic treatments in clinical care or low-dosage intake from food could be contributing factors in this issue. Researchers in both human and animal studies have strived to explain this multifaceted relationship. The present review intends to elucidate the axis of the gastrointestinal microbiota and antibiotics resistance and to highlight the main aspects of the issue.

## 1. Introduction

Undoubtedly, since the discovery of penicillin by Alexander Fleming in 1928 and thereafter, antibiotics used in the management of infectious diseases have saved millions of lives [1]. Over the past two decades, antibiotic misuse and overuse has come to be considered a serious public health issue, imperiling the great achievements of medicine. Antimicrobial resistance (AR) is a developing concern that threatens to harm the effective treatment of infectious diseases, especially in high-income countries [2]. Even though the irrational use of antibiotics was once considered a problem only in developed countries, a striking rise in low and middle-income countries has occurred [2]. It is of interest to note that de Jong et al., in their observational study, revealed that antibiotic-resistant bacteria from animal farms could be the reason for therapeutic failure in adults living in rural areas, an assumption that of course needs further investigation [3]. However, another study described that higher prevalence rates of 8-fold were observed in urban settings when compared to rural settings, as antibiotic prescription was more frequent in towns with hospitals. The antibiotic prescription rate in urban areas was 46.8% where people receive more qualified hospital care [4]. AR occurs more frequently in hospitals due to the increasing number of patients, surgical procedures, and interventions, which are linked to the increasing use of antibiotics in the health care setting.

Antibiotics resistance percentages are rising daily, not only concerning the hospital community, but also various other territories. Antibiotics are given to animals for treating infections, but mostly to achieve faster growth for commercial purposes. Moreover, AR is also present in plant pathogens [5]. Antibiotics used for therapy and animal feeding contribute to the spreading of antibiotics resistance in food and environment [6]. Moreover, ESKAPE (*Enterococcus*, *S. aureus*, *K. pneumoniae*, *A. baumannii*, *P. aeruginosa*, and *E. coli*) pathogens are a major cause of hospital-acquired resistant infections worldwide, as they are associated usually with more serious morbidity and mortality rates and consequently to an important economic loss due to various complications, prolonged hospitalization, expensive drugs, and absenteeism in workplaces [5].

It is well known that antibiotics can produce alterations in the host’s indigenous microbiota by selecting resistant bacteria that can appear as opportunistic pathogens [7]. Additionally, a low-dosage intake of antibiotics or sub-therapeutic antibiotic treatment (STAT) from food and the environment have also been associated with gut dysbiosis. Gut dysbiosis promotes negative effects in plenty of systems and functions of the host. Since the gut microbiome could be “at the intersection of everything”, its alterations have been linked to multiple pathological conditions, and scientists have focused on the relationship between antibiotics and the gut microbiota [8]. Accumulating evidence mainly from animal studies has underscored the contribution of antibiotics to gut microbiome disruptions [9,10]. Although morbidity and mortality, due to infectious diseases, were remarkably reduced, antibiotic treatment has been implicated in gut microbiota disruptions.

Nowadays, resistance represents a common trait for almost all developed antibiotics. Unfortunately, at the end of 20th century the development of new antibiotics was dramatically decreased due to economic and regulatory obstacles [11].

In this review, we summarize current evidence regarding the gut microbiome and its alterations in relation to antibiotics, analyzing the reasons associated with their inappropriate use.

## 2. Insights to the Gut Microbiome

Until now, the usage of classic microbiological techniques has limited the amount of information found about the human microbiome. However, the introduction of new molecular methods such as next-generation sequencing (NGS) and methodologies such as 16S ribosomal RNA (rRNA) gene sequencing and metagenomic shotgun sequencing have revolutionized scientists’ knowledge about these microorganisms.

The abundance, diversity, and features of microorganisms’ genes are collectively known as the human microbiome, a seemingly “new actor on stage” due to its numerous roles in health and disease [12]. Several publications have demonstrated the relationship between dysbiosis and inflammatory and metabolic diseases, such as inflammatory bowel disease (IBD), obesity, cancer, asthma, autism, autoimmune diseases, etc. [13]. Until now, these studies have failed to establish a causative role for the microbiome but have mainly focused upon the relationship between pathogenesis, clinical manifestations, and disease prognosis with microbiome alterations.

The human microbiome is comprised of almost 40 trillion bacterial cells and about 30 trillion human ones, revising the notion of the ratio closer to 1:1 [14]. Most microbes belong to five major phyla: *Firmicutes*, *Bacteroidetes*, *Actinobacteria*, *Proteobacteria*, and *Verrucomicrobia* [15]. The gut holds the majority of species—around 2000, with *Bacteroidetes* and *Firmicutes* representing more than 90% of its microbes [16,17]. The gut microbiome contributes to human body functions such as digestion, metabolism, protection from pathogenic microbes, the production of vitamins, as well as the regulation of the immune system and inflammatory reactions. These functions represent those of an “active organ” [7] or a microbial “endocrine organ” [18,19].

The human gut microbiome has been categorized into three enterotypes according to the variation in gut microbes [20,21,22]. A person’s enterotype could change due to different factors such as gender, age, food intake, vaccinations, infections, smoking, etc., resulting in differences in the composition and diversity of the gut microbiota from newborns to elders [7,23]. The gut is massively colonized after birth, excluding the possibility that the fetal gut is sterile [24,25,26]. Moreover, it was shown that the composition of the human microbiome is affected by age and comorbidities [27]. A “healthy” gut microbiome has a high diversity; any kind of disruption may lead to dysbiosis, a critical condition of imbalance between commensal and pathogenic microbes [28]. Eubiosis due to beneficial bacteria maintains an important homeostatic niche by preventing any disequilibrium that might cause dysbiosis and, consequently, metabolic and inflammatory conditions, including asthma, obesity, cancer, autism, and autoimmune diseases [10].

As mentioned, a specific diet may shape the profiles of gastrointestinal bacteria in humans. Differences in food intake create a different community structure of the gut microbiota [7]. For example, comparing the microbiota of European children (EU) to children coming from a rural African village in Burkina Faso (BF), an environment that resembles that of Neolithic farmers, by high-throughput 16S r DNA sequencing and biochemical analysis [29] one could see that BF children presented a higher proportion of *Bacteroidetes* numbers than *Firmicutes*. Moreover, *Prevotella* spp. and *Xylanibacter* spp. were prevalent in these children; both are involved in cellulose and xylan hydrolysis. However, these bacteria were absent from the intestinal microbiota of the EU children. Additionally, the BF children showed a higher ability to produce metabolites such as short-chain fatty acids (SCFAs). The microbiota of BF and EU children has co-evolved with diet since ancient times, and the high amounts of SCFA seemed to provide the host with an important amount of energy [25,26]. In both populations, Actinobacteria with a predominance of the genus *Bifidobacterium* were present in younger infants who were breast-feeding [7,26].

The characteristic profile of the newborn gastrointestinal microbiota depends on age, race, and the subject’s diet [30,31,32,33,34]. Several hours after birth, the newborn develops its normal microbiota. Colonization by *Bifidobacterium* happens within four days after birth. Breast-fed infants carry a typical gut flora featuring an increased concentration of *Bifidobacterium*. However, infants receiving artificial alimentation do not usually carry *Bifidobacterium* or demonstrate low concentration numbers, showing a generally lower microbial diversity. Moreover, male newborns show a higher count of *Bifidobacterium* than females. Nevertheless, in both sexes its preponderance is manifested after maternal alimentation. Positive effects of *Bifidobacterium sp*. on infant growth and health status have been reported [7]. A fierce competition has been exhibited between *B. bifidum* and *C. perfringens* in the gut of newborns delivered by caesarean section [7,34]. Multiple authors have stated the beneficial action of several bacteria on the intestinal ecosystem, amongst them *Bifidobacterium* spp. [35,36,37,38].

By the time the child reaches the age of three or four years, two dominant phyla exist: *Firmicutes* and *Bacteroidetes*. The *Firmicutes* phylum includes *Lactobacillus*, *Bacillus*, *Clostridium*, *Enterococcus*, and *Ruminicoccus* genera, which may exhibit diametrically opposite actions. For example, *Faecalibacterium prausnitzii* is more abundant in the gut of obese children than in non-obese children, whereas *Clostridia* in human feces are associated with s lower body mass index. Moreover *E. feacalis* escaping from the gut might cause a deleterious blood infection, while *Bacteroidetes* are protective [18,19,39,40]. However, the differences were related to the presence of the *Firmicutes* phylum’s class, the *Mollicutes* class, as obtained by animal studies with diet-induced obesity [41]. From this point forward, the gut microbiota tends to maintain a well-balanced condition, with few changes across the adult life, ending in a different state in the elderly [42], who show a decrease in *Bifidobacterium* spp. [38,43]. Diet and drugs correspond to critical microbiome alterations, when other factors, such as genetics, have less impact on the microbial population [42].

In the human intestine, bacterial levels rise along the intestinal lumen. For example, the bacterial numbers can be as high as 10 million bacteria/mL in fecal fluid. Qualitative and quantitative differentiation is registered in bacterial populations colonizing different parts of the gastrointestinal ecosystem [18]. *Lactobacillus*, which are facultative anaerobic or aerobic rods, are permanent residents of the ecosystem of the human gut [18,37]. Different studies suggest that the advantageous effects of Lactobacillus are strain-dependent. Agerholm-Larsen L et al. reported weight gain with the use of *L. rhamnosus* and also with L. acidophilus [44]. On the other hand, *L. gasseri BRN17* and *L. gasseri SBT2055* in different studies are associated with weight loss [45]. *Lactobacillus* show a selective adherence to the intestinal epithelial cells [46,47]. *Enterobacteriaceae* are associated with gastrointestinal infections and carry specific adhesins, which mediate their adhesion to the intestinal mucosa [48,49]. Therefore, non-pathogenic anaerobic bacteria, such as *Lactobacillus* and *Bifidobacterium*, could impede the ability of the adhesion and invasion of several enteropathogenic enterobacterial strains [49].

In addition to food-induced effects on the gut microbiome, a significant contribution to its development is derived from the administration of probiotics, prebiotics, and antibiotics [50]. Probiotics and prebiotics might offer a more balanced protection in the gut, but antibiotics might decrease diversity and promote dysbiosis [51,52,53]. Another factor that might decrease diversity and promote dysbiosis is alcohol abuse [54]. Nevertheless, the explicit factors defining the development of beneficial lactic acid microbiota are not perfectly clarified, but research focusing on the distributions of different strains in the various human organs, during states of health and disease, may elucidate them. Adequate knowledge of the intestinal microbiota and its probiotic profile in health and disease could provide therapeutical advancements. Therefore, the probiotic approach will assist the investigation of the role of bacterial species, as well as those components promoting their growth in the human intestine.

## 3. Antibiotic-Associated Shifts in the Gut Microbiota

As soon as antibiotics were introduced, they were acknowledged as the most effective and life-saving drugs to combat infectious diseases, and they resulted in a substantial decrease in morbidity and mortality. However, humanity soon realized that the irresponsible and thoughtless use, misuse, and overuse of antibiotics led to the emergence of antimicrobial resistance (AR). AR poses a global threat to modern medicine and its achievements and is a major health problem [55,56]. Additionally, recent studies have illuminated the potential impact of antibiotic intake on the intestinal microbiome. Antibiotics can negatively affect the required diversity of the gut microbiota in adults [57,58,59,60] and children [61]. The short-term effects of antibiotic use include diarrhea, *Clostridium difficile* infection, and AR [62,63,64], whereas the long-term consequences include the development of allergic conditions—namely, asthma or food allergies and obesity [65,66].

Antibiotic administration for therapeutical purposes affects the bacterial microbiota both quantitatively and qualitatively by reducing or eliminating bacterial species and allowing other species to obtain more space and nutrients in the intestine. This microbial imbalance influences the state of health and disease. However, these studies have faced limitations, such as drug composition and route of administration, as well as the age of the patient, the deleterious impact of antibiotics in early life [60], and other factors such as diet and functional foods [67,68,69,70]. In particular, Cox et al. introduced the concept of the “critical developmental window” in the early life of mice when low-dose antibiotics had the greatest impact on the gut microbiome, leading to metabolic effects [71].

The impact of antimicrobial agents used therapeutically or as a prophylaxis on normal gastrointestinal microbiota causes disturbances in the ecosystem’s equilibrium. In all cases, disequilibrium and alterations in the microbiota ecology depend on the involved drug and its pharmacokinetic profile [72]. The human intestine has the capacity to metabolize drugs due to the possession of an enormous carriage of Cytochrome P450 (CYP) enzymes, which are responsible for the catalyzation reactions in phase I and phase II of drug metabolism [72]. Korpela et al. have demonstrated that oral antibiotic therapy with macrolides led to changes in the intestinal microbiota by creating a shift in the relative abundance of *Bacteroides* and *Bifidobacterium* [73]. In the same vein, antibiotic treatment breaks the intestinal equilibrium, leading to a niche perfect for *C. difficile* growth and spore germination [74,75]. However, other authors have stated an antibiotic-induced rise in toxin production by *C. difficile* as a stress-induced response that may vary following the bacterial strain [76].

Likewise, antibiotic abuse lead to negative effect on the levels of proliferation or apoptosis of intestinal cells, to enterocytes (sucrase) and endocrine cells. Therefore, lots of intracellular proteins are released. Except for the local functional activity of releasing proteins, they may be useful as markers of gut microbiome dysregulation. Zhernakova et al., in their study of the gut microbiota of 1135 participants from a Dutch population-based cohort, demonstrated a connection between the microbiome and the different host factors. Authors reported that fecal chromogranin A (CgA) was exclusively associated with the presence of particular microbial species [77].

### Immunomodulatory and Indirect Effect of Antibiotics on the Gut Microbiota

The effect of antibiotic drugs to the human microbiome is complex and bi-directional. Except for direct effect, antibiotics can also indirectly affect human microbiota. The gut microbiota dysbiosis following exposure to antimicrobial agents may cause the dysregulation of immune responses [78]. Indeed, it was demonstrated with in vitro and ex vivo studies how a short-term treatment with broad-spectrum antibiotics deeply affected both cellular and humoral immune response [79,80,81]. Some antibiotics have been reported to display immunomodulatory effect in addition to their antimicrobial activity [82,83,84]. Konstantinidis et al. demonstrated that macrolides such as clarithromycin can induce Neutrophils Extracellular Trap (NET) generation both in vitro and in vivo. Moreover, in this study the authors showed that clarithromycin-induced NETs are decorated with functional antimicrobial peptide LL-37, which is able to inhibit the growth of multidrug resistant strains [84]. In addition, LL-37 plays a critical role in the protection of the colon microbiota balance [85]. Di Fan et al. found that hypoxia-inducible factor-1α (HIF-1α), a transcription factor for human cathelicidin (LL 37), is important for activating innate immune effectors and is the key determinant of *Candida albicans* colonization resistance [86]. Moreover, LL-37 plays multiple roles in innate immune responses and wound healing [87,88]. Yoshimura et al., in their ex vivo model of CRAMP−/− mice, showed that CRAMP−/− mice developed more severe colitis and succumbed rapidly [87]. Furthermore, Inomata et al. reported than the antimicrobial peptide LL-37 upregulates the expression of several immune-related genes [89]. The authors investigated the effect of LL-37 on the gene regulation of human gingival fibroblasts (HGFs). During this study, it was proven that LL-37 alters the expression of 29 genes that encode TLR-associated proteins. Moreover, LL-37 increased the LPS-upregulated expression of IRAK1 [89]. Apart from the well-documented mechanisms related to LL-37 effects on neutrophils and monocytes, T-cells also respond to LL-37 stimulation via T-cell proliferation, T-cell activation, as well as the generation of regulatory T-cells (Tregs) [90]. Human antimicrobial peptides are abundantly secreted by colonic epithelial cells and are critical components of innate immune response against enteropathogenic bacteria such as *Shigella* spp., *Salmonella* spp., and *C. difficile*. The antibiotics-induced synthesis of AMPs is the cornerstone mechanism of the indirect action of this group of drugs on the human microbiome.

By the same token, antibiotics’ influence on intestinal bacterial diversity and long-term abuse has been identified as an independent risk factor for metabolic disorder, such as atherosclerosis-driven events. Kappel et al., in their experimental animal model, showed that the augmented atherosclerosis induced by antibiotics was correlated to a loss of gut microbiome’s diversity by a reduction in *Bacteroidetes* and *Clostridia* [91]. Moreover, antibiotics as gut microbiome modulators alter the immune response to various non-infectious diseases and drugs, such as immune checkpoint inhibitors (ICI) in patients with solid neoplasms. Kapoor et al. reports that the median overall survival of the patients who received antibiotics in this window was 2.8 months (95% CI: 1.2–4.5) as compared to 9.2 months (95% CI: 5.2–13.1) in those who did not receive antibiotics *p* = 0.008 [92].

Antimicrobial agents induce autophagy and the inhibition of the immune response. In this context, antibiotics may alleviate the progression of the autoimmune and neuroinflammatory diseases [93]. Studies show that antibiotics may influence the pathogenesis of neurodegenerative diseases, such as multiple sclerosis and Amyotrophic Lateral Sclerosis (ALS), through gut microbiome dysfunction [94,95,96]. Some antibiotics, such as beta-lactam, except for direct antimicrobial effects also act as neuromodulators due to the upregulation of the glutamate transporter 1 (GLT-1) expression [97].

Previous studies have shown that the microbiome plays a critical role in chemotherapy-induced peripheral neuropathy (CIPN) [98,99,100]. Ramakrishna et al. report that chemotherapeutic agents cause barrier dysfunction, resulting in increased systemic exposure to bacterial products and metabolites, which promote both local and systemic inflammation, which drive pain sensitivity. The authors believe that microorganisms *Porphyromonadaceae* are associated with both bacteria and pain as well as between microglia and pain, and that gut bacteria modification by antibiotics has a positive effect on this phenotype [100].

Another pathway of the indirect effect of antibiotics on the human microbiome is the regulation of radical nitric oxide (NO) synthesis by the activation of the inducible nitric oxide synthase. NO increases mucosal blood flow and mucus thickness and prevents microbial infections [101]. However, the impact of NO on the gut microbiota remains elusive. Studies indicate that the NO plays a vital role in host defense against bacterial infections [102].

**Figure 1:**
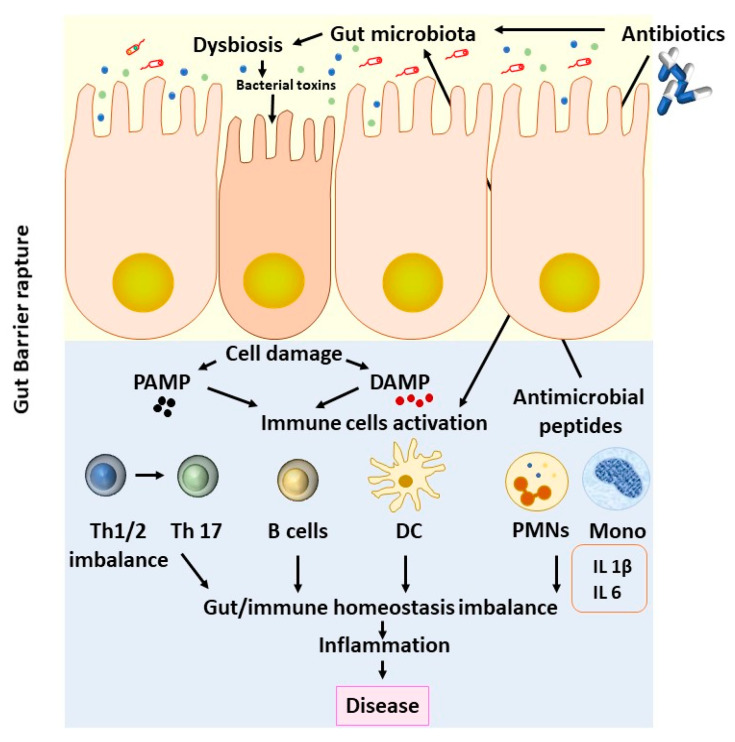
Effects of antibiotics upon the gut microbiome. Antibiotic treatment is crucial for combating infections. On the other hand, antibiotic exposure can alter many basic equilibria in terms of intestinal microbiota and host immunity, promoting long-term disease. DC: dendritic cells; DAMP: damage-associated molecular patterns; PMNs: polymorphonuclear leukocytes; PAMP: pathogen-associated molecular patterns; Th: T helper cells.

Immune cells play a significant role in the maintenance of tissue homeostasis by exhibiting the plasticity of their phenotypes, such as M1 or M2 for macrophages or N1 and N2 for neutrophils. Microbiota-derived metabolites, short-chain fatty acids (SCFAs), bacterial lipopolysaccharides (LPS), and antimicrobial peptides wield anti-inflammatory or pro-inflammatory effects by acting on immune cells [103,104]. Maekawa et al. demonstrated that the anti-inflammatory action of erythromycin is mediated through the upregulation of the secreted homeostatic protein DEL-1 [105]. Through this study, it was shown that erythromycin regulates neutrophil function in the tissues, such as lungs or the periodontium, in a DEL-1-dependent manner (Figure 1).

A high-fat diet (HFD) exhibited impaired neutrophil migration to the intestinal mucosa and reduced the gene expression of the CXCL-1 chemokine and CXCR-2 receptor in the ileum [106]. In this context, it was previously shown that the depletion of neutrophil migration is also correlated with the proliferation of tumor cells and tumor-cell DNA damage in an interleukin-17-dependant manner [107]. Moreover, a high-fat diet induced neutrophil activation by enhancing neutrophil elastase activity [108]. The high levels of active neutrophil elastase are associated with a low microbiome diversity and the downregulation of microbiome characteristics [109]. In addition, neutrophil extracellular traps (NETs), as cornerstone mechanisms of neutrophil action, are involved in several disease exacerbations. Dicker et al. report that NETs are associated with disease severity in patients with Chronic Obstructive Pulmonary disease (COPD), a loss of microbiota diversity (*p* = 0.009), and the dominance of *Haemophilus* species’ operational taxonomic units (*p* = 0.01) [110]. Besides this, it was previously reported than neutrophil ageing is regulated by the microbiome. This mechanism is driven by the microbiota via the TLR receptor and myeloid differentiation factor 88-mediated signaling pathways [111].

## 4. The Reservoir of Antibiotics in Animal Feed and the Emerging Resistome

### 4.1. AR in the Food Chain

Due to the increased development of animal production in industrial plants, antibiotics are added to feed for the efficient feeding of animals and poultry and for improving their growth. From the total amount of produced antibiotics, 40% are used for this purpose [112]. In Europe, their use has been registered since 1953. Penicillin and tetracyclines for low-level feeding (5 to 10 g/ton) are used in premixes or feed supplements as growth promotions, specifically in poultry. A plethora of other antibiotics, such as swine and ruminants, is used for producing meat. In addition to animal growth, these antibiotics aid in the reduction in enterotoxaemia symptoms. The use of antibiotics for growth promotion purposes was banned in the European Union in 2006 (European Commission, Brussels (December 2005): “Ban on antibiotics as growth promoters in animal feed enters into effect”). Similar actions were taken as of 2017 in the U.S.A. for drugs that are important to human health [113].

Antibiotics are purchased from the feed industry or from veterinary supply centers and are given to animals, usually by being placed in their drinking water. In the United States, farmers use more than 17 antibiotics in animal husbandry [114]. The amount of antibiotics used for infections is four times less than the quantities used for breeding livestock, as the Food and Drug Administration (FDA) stated in 2011 [115]. On 18 December 2018, the FDA reported that “domestic sales and distribution of all medically important antimicrobials intended for use in food-producing animals decreased by 33 percent between the years 2016 and 2017”, probably due to effective antibiotic stewardship [116].

Antibiotics use also provides a clear benefit for the producer, as less feed is needed for the animal to achieve the desired weight development; therefore, the cost of purchasing food for the animal is reduced. Nevertheless, the mechanism of antibiotic use as a growth promoter is not yet clarified. Animals are believed to develop latent infections following the production of catabolic products and cytokines that interfere with the growth of animal flesh due to unhygienic conditions during breeding. Antibiotics can prevent this situation by suppressing pathogens [117]. Alternatively, animal feed is never sterile. In this vein, bacteria grow by consuming nutrients found in feed and producing toxic substances that have adverse outcomes on animal health. Therefore, antibiotics overcome these harmful bacteria in animal feed [117]. However, the use of antibiotics in this way must be banned due to the increasing problem of AR. The administration of antibiotics leads to the development of AR, which seems to be associated with the extended use of antibiotics rather than their short-term use [112]. Therefore, low-level antibiotic feeding causes bacterial resistance [118,119]. Antibiotics misuse in both animals and humans leads to a significant increase in antibiotic-developed resistance [120,121], and this resistance can be transferred through plasmids from resistant bacteria to sensitive ones [122]. Moreover, hazards associated with animal health from using low-level antibiotics include the development of resistant pathogenic strains, as well as increasing susceptibility to several infections due to the disturbance of the microbiota or to immunosuppression [7]. Promising antimicrobial agents have been developed and could be used in the animal industry, whilst effective vaccines are available for enterotoxaemia and other infectious diseases. Should an animal vaccination program be introduced, the constant demand for disease surveillance through antibiotics could wane, as long as research is progressing constantly in this direction.

Microbial communities survive in highly antagonistic environments where the nutritional sources available can define their growth and genetic persistence. Human activities select resistant strains and strengthen the transfer of genetic information from unlinked bacterial species by creating environmental niches [123]. Antibiotic resistance is also developed in plant pathogens [124]. Furthermore, domestic, hospital, and industrial waste contributes to the selection of resistant strains. Thus, resistant bacteria can be passed onto other hosts in different ways, or their mutations can be passed to subsequent bacterial generations. As stated, environmental niches (pathogenicity islands) that carry multiple drug-resistant genes can be formed.

Researchers have performed studies in humans under clinical treatment or experimental exposure (volunteers) to antibiotics [43]. Additionally, other researchers have investigated the different functions of the intestinal microbiota subsequent to antibiotic administration in germ-free animals [125]. The importance of the ecological equilibrium of the intestine, called “colonization resistance”, as antibiotic resistance is spreading between humans, should be limited [126]. Apparently, antimicrobials entered the food chain a long time ago, and human existence has already been continuously influenced for a significant amount of time. The use of antibiotics as growth promoters is suspected to be a contributing factor in the emergence of resistant microbial strains responsible for detrimental infectious diseases.

### 4.2. AR Genes in the Intestinal Microbiome

Although the gut microbiome may be considered as the basis of the host’s wellbeing, at the same time it creates potential threats due to the presence of ARgenes (antibiotic resistance genes). It could be a “reservoir” of Multi Drug Resistant Bacteria (MDR) or Pan Drug Resistant Bacteria (PDR) and their antibiotics resistance genes [127]. Taken together, the ARgenes and their ancestors of pathogenic and non-pathogenic gut bacteria comprise the “resistome”, as proposed by Gerard D. Wright in 2006 and 2007 [128,129]. Scattering ARgenes by different methods—namely, horizontal gene transfer, toxin–antitoxin systems, and Mobile Genetic Elements (MGEs)—creates a huge reservoir of AR determinants in the intestinal microbiome. However, it seems that the majority of these determinants are considered innate and are not shared with opportunistic pathogens [130]. Furthermore, the “mobilome”, which consists of MGEs, serves as a path for transferring ARgenes among intestinal bacteria [127]. Metagenomic research revealed that, after extended antibiotic treatment, especially with aminoglycosides, an augmentation in the relative abundance of ARgenes emerged [131,132]. *Clostridium difficile* is a well-known factor causing nosocomial diarrhea because of prolonged broad-spectrum antibiotic treatment, and it is worth stating that probiotics (beneficial microbes for the gut) together with antibiotics might prevent clinical infections [133]. Probiotic bacteria as well as dietary interventions could be very promising, either preventing undesired shifts in the gut microbiome due to antibiotics or restoring the harmed balance after detrimental antibiotic use [127,134,135].

## 5. Conclusions

Antimicrobial resistance poses an immense threat to global health. There is a considerable amount of evidence from animal models regarding the involvement of disrupted intestinal microbiota under antibiotic treatment. The role of antibiotics as a catalyst in this interaction, either as therapy or through low-dosage intake through the food-chain, has not yet been fully clarified. New cutting-edge techniques and more sophisticated and randomized control trials are required to elucidate the relationship and examine the potentials and challenges for combating the new epidemic of AR. The World Health Organization (WHO), following an extended surveillance study of antimicrobial resistance, evinces the severity of the problem and emphasizes the necessity of concerted action among all states and involved bodies in order for society to mitigate antimicrobial resistance’s colossal threat. Besides this, the economic losses linked to antimicrobial resistance should be noted. In this vein, global collaboration between scientists will permit us to explore and establish the best policy and the most effective process and strategy in the community and the environment.
